# Supplementary material for: Field-resilient supercurrent diode in a multiferroic Josephson junction
Source: Nat Commun. 2025 Oct 21;16:9287. doi: 10.1038/s41467-025-63698-3 (PMC12540659; doi:10.1038/s41467-025-63698-3)
Supplement: Supplementary file 1 — Supplementary Information [file 41467_2025_63698_MOESM1_ESM.pdf]

Supplementary information for

Field-Resilient Supercurrent Diode in a Multiferroic Josephson Junction

Hung-Yu Yang[1*], Joseph J. Cuozzo[2,3], Anand Johnson Bokka[1,4], Gang Qiu[1], Christopher Eckberg[1], Yanfeng Lyu[5], Shuyuan Huyan[6], Ching-Wu Chu[6,7], Kenji Watanabe[8], Takashi Taniguchi[9] and Kang L. Wang[1*]

[1]Department of Electrical and Computer Engineering, University of California, Los Angeles, CA, USA.
[2]Materials Physics Department, Sandia National Laboratories, Livermore, CA, USA.
[3]Department of Physics, The University of Texas at El Paso, El Paso, TX, USA.
[4]Department of Materials Science and Engineering, University of California, Los Angeles, CA, USA.
[5]School of Science, Nanjing University of Posts and Telecommunications, Nanjing, China.
[6]Department of Physics and Texas Center for Superconductivity, University of Houston, Houston, TX, United States.
[7]Lawrence Berkeley National Laboratory, Berkeley, CA, United States.
[8]Research Center for Electronic and Optical Materials, National Institute for Materials Science, Tsukuba, Japan.
[9]Research Center for Materials Nanoarchitectonics, National Institute for Materials Science, Tsukuba, Japan.

*Corresponding author(s). E-mail(s): hungyuyang@ucla.edu; wang@ee.ucla.edu;

Table of Contents

1 Schematics and optical images of the reported superconducting devices. . . . 3
2 Robust supercurrent switching in the NiI_2 device. . . . 4
3 Inelastic electron tunneling experiments on the graphite/NiI_2/graphite tunnel junction. . . . 5
4 Fraunhofer interference patterns of Gr JJ and NiI_2 JJ. 6
5 Out-of-plane magnetic field dependence of supercurrent diode effect in the NiI_2 JJ. . . . 7
6 Temperature dependence of the resistance across the junction and flakes. . . . 8
7 Additional simulations of a helimagnet JJ. . . . 9
8 Simulation of a wide helimagnet JJ. . . . 10
9 Simulations of multiferroic JJs. . . . 11
10 Single-ℓ helical superconductivity with a helimagnet. . 12

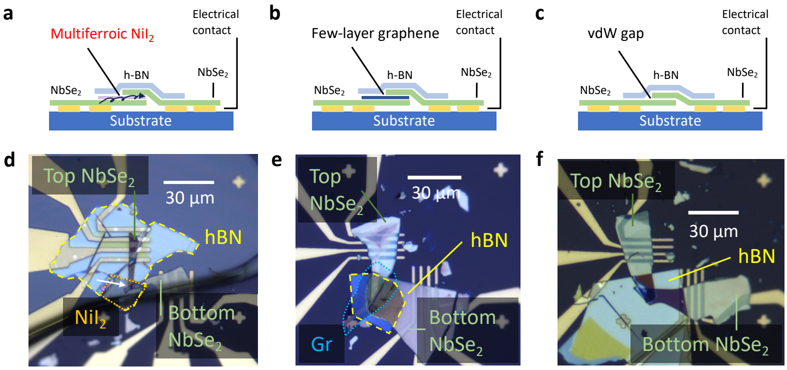

Supplementary Fig. 1 Schematics and optical images of the reported superconducting devices. a-c, Side views of NiI_2 JJ, Gr JJ, and $NbSe_2/NbSe_2$ devices. **d-f**, Optical images of NiI_2 JJ, Gr JJ, and $NbSe_2/NbSe_2$ devices. The junction area of NiI_2 JJ with a lateral dimension of 2-3 μm is pointed out by the white arrow. The thickness of the following flakes was measured by AFM: NiI_2 in panel d: 2.8(2) nm (4ML); few-layer graphene in panel e: 4.0(2) nm. The thickness of the following flakes is estimated from the thickness of the flakes with similar color contrast measured by AFM: top $NbSe_2$ in panel d: $\sim$20 nm; bottom $NbSe_2$ in panel d: 30-40 nm; top $NbSe_2$ flake in panel e: $\sim$20 nm; bottom flake in panel e: 60-70 nm; both $NbSe_2$ flakes in panel f: $\sim$30 nm. Multiple colors can sometimes be seen in a single flake, and the thickness above provided the best estimation of the thickness near the junction area.

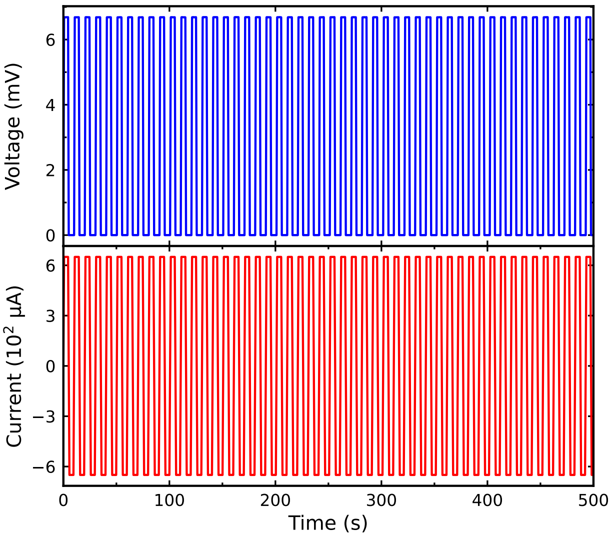

Supplementary Fig. 2 Robust supercurrent switching in the NiI_2 device. Demonstration of supercurrent switching with $I_{\text{bias}} = \pm 650\ \mu$A used in Fig. 1d of the main text for 50 cycles.

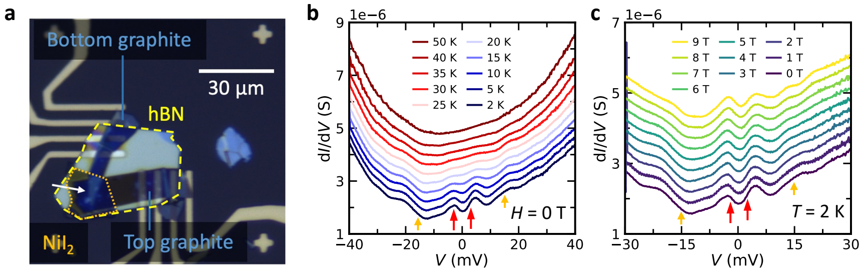

Supplementary Fig. 3 Inelastic electron tunneling experiments on the graphite/NiI_2/graphite tunnel junction. **a**, The optical image of the graphite/NiI_2/graphite tunnel junction device. The junction area is pointed out by the white arrow. The thickness of the NiI_2 flake at the junction is 10.5 nm as measured by AFM. The thickness of the top and bottom graphite flakes is about 20 nm and 40 nm based on the AFM results of other flakes of similar color contrast. **b**, Tunneling conductance v.s. dc voltage bias measured at different temperatures at zero field. The data taken at temperatures other than 2 K are shifted vertically for clarity. The red arrows ($\sim \pm$ 3-5 meV) and orange arrows ($\sim \pm 15$ meV) represent a symmetric step-like increase of tunneling conductance, potentially enhanced by collective excitations [1]. The inferred excitation energies agree with the electromagnons and magnons energies in NiI_2 as detected by Raman spectroscopy and Terahertz spectroscopy [2, 3]. The onset temperature of collective excitations agrees with the ordering temperature of NiI_2 in the thin layer limit, supporting their magnetic origin [2]. **c**, Tunneling conductance v.s. dc voltage bias measured at different in-plane magnetic fields. The data taken at fields other than 0 T are shifted vertically for clarity. The features representative of collective excitations in NiI_2 persist all the way to $H_{\|} = 9$ T, suggesting that the multiferroic order in thin-layer NiI_2 survives to high magnetic fields as in the bulk limit [4].

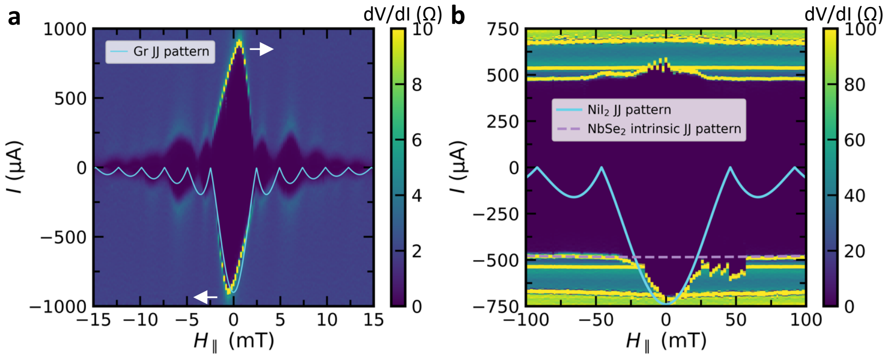

Supplementary Fig. 4 Fraunhofer interference patterns of Gr JJ and NiI_2 JJ. **a**, Critical current mapping of the Gr JJ at $T = 2$ K. The white arrows point out the tilt of the central maxima towards opposite directions due to the self-field induced by the device's cross-junction geometry (see Fig. 1 of the main text and Supplementary Fig. 1) [5–7]. The supercurrent flowing throguh the top and bottom $NbSe_2$ flakes generates current-dependent magnetic field (H_{self}) at the few-layer graphene weak link in addition to the externally applied magnetic field. In this case, the maximum of I_c appears at a finite magnetic field (instead of zero field) with which H_{self} is offset, causing the shift of maxima and the tilt of the interference pattern. The tilted pattern is directly linked to the anti-symmetric component of SDE in Gr JJ and NiI_2 JJ shown in Fig. 2 of the main text. Other than the tilt, the field dependence of the critical current agrees with the Fraunhofer interference pattern as expected from a dc Josephson effect, $I_c(H) = I_c(0)|\sin(\pi\Phi/\Phi_0)/(\pi\Phi/\Phi_0)| = I_c(0)|\text{sinc}(\Phi/\Phi_0)|$, where Φ_0 is the magnetic flux quantum, and Φ is the magnetic flux going through the JJ [8]. Typically, Φ is calculated as $H_\parallel \times (d + 2\lambda) \times W$, where d is the separation between the two superconducting electrodes, λ is the London penetration depth of the superconductor, and W is the lateral width of the junction. Here, we use $\Phi = H_\parallel \times d_{\text{eff}} \times W$ to model the interference patterns in our vdW JJs. With W determined from the optical image of the device, the simulated curve (cyan line) showing a good agreement is generated with $d_{\text{eff}} = 40$ nm, similar to other vdW JJs reported in the literature [9–11]. We also note that if the flux focusing effect [12] plays a role, a slightly modified expression $I_c(H) = I_c(0)|\text{sinc}(\Gamma\Phi/\Phi_0)|$ with $\Gamma = 3$ and $d_{\text{eff}} = 14$ nm can also lead to a decent agreement between the observed and simulated patterns. **b**, Critical current mapping of the NiI_2 JJ at $T = 2$ K. At low fields, a typical interference pattern ($I_c(0)|\text{sinc}(\Phi/\Phi_0)|$) as expected from the NiI_2 JJ was observed (cyan solid line, $W = 3$ μm and $d_{\text{eff}} = 15$ nm). At high fields, the pattern was truncated by the other interference pattern caused the interlayer Josephson coupling between each layer within $NbSe_2$ (purple dashed line). The coexistence of both types of interference patterns was also observed in artificially engineered multilayer JJ [13] and twisted $Bi_2Sr_2CaCu_2O_{8+x}$/$Bi_2Sr_2CaCu_2O_{8+x}$ vdW JJ [14] as a result of two JJs in series. In general, in vdW JJs, the Josephson coupling may established between 1) the vdW gaps within each superconducting flake, and 2) the weak link between the top and bottom flakes. We note that in our work, such a behavior is present in the NiI_2 JJ but not in the Gr JJ, most likely due to the difference of Josephson penetration depths λ_J (~ 10 μm for the NiI_2 JJ and ~ 400 μm for the Gr JJ) compared to the junction's lateral width w (about several μm) [15]. The huge difference between the λ_J of Gr JJ and w makes the Josephson coupling across the few-layer graphene the dominant one, while the proximity between λ_J of NiI_2 JJ and w makes it less dominant and potentially comparable with the intrinsic Josephson coupling within $NbSe_2$ flakes, creating a two-JJs-in-series scenario and the coexistence of two interference patterns.

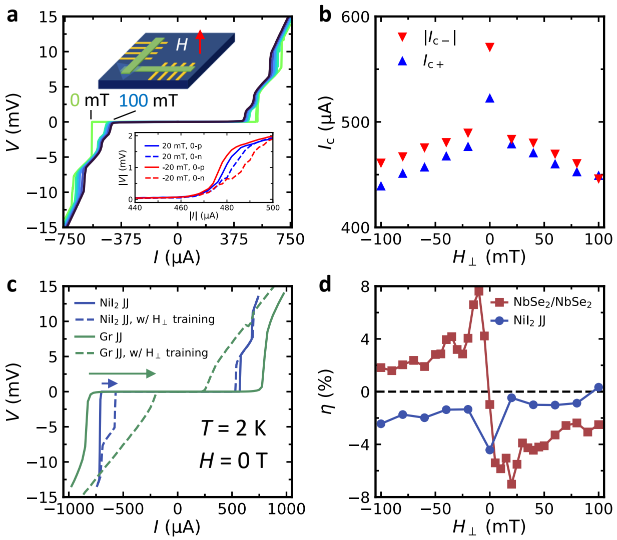

Supplementary Fig. 5 Out-of-plane magnetic field dependence of supercurrent diode effect in the NiI_2 JJ. **a**, $V - I$ characteristic of the NiI_2 JJ with 0 mT $< H_\perp <$ 100 mT, with a 20 mT field increment at $T = 2$ K. Inset: Representative switching curves at $H_\perp = \pm 20$ mT showing negative supercurrent diode effect in both cases. **b**, Critical current I_{c+} and $|I_{c-}|$ as a function of $H_\perp$. At zero field, it is noted that the ΔI_c as deduced from panel b is smaller than the corresponding zero-field value from Fig. 2b of the main text, due to the reduction of critical current when the measurement is taken after an out-of-plane field history. **c**, $V - I$ characteristic of NiI_2 JJ and Gr JJ with and without an $H_\perp$ training history. The short blue and long green arrows show the reduction of I_{c-} after an $H_\perp$ training for NiI_2 JJ (-100 mT) and Gr JJ (-10 mT), respectively. The reduction is likely due to trapped Abrikosov vortices induced by $H_\perp$ in the $NbSe_2$ electrodes [**?**], different from the Josephson vortices induced by $H_\parallel$. **d**, Comparison of η as a function of $H_\perp$ between the $NbSe_2/NbSe_2$ junction and the NiI_2 JJ. The $NbSe_2/NbSe_2$ homostructure can be viewed as a superconductor with enhanced inversion symmetry breaking by stacking two $NbSe_2$ flakes. It shows an SDE with anti-symmetric out-of-plane field dependence, as expected from superconductors without inversion symmetry [17]. The NiI_2 JJ defies this general trend and shows a predominantly symmetric field dependence with a persistent negative η.

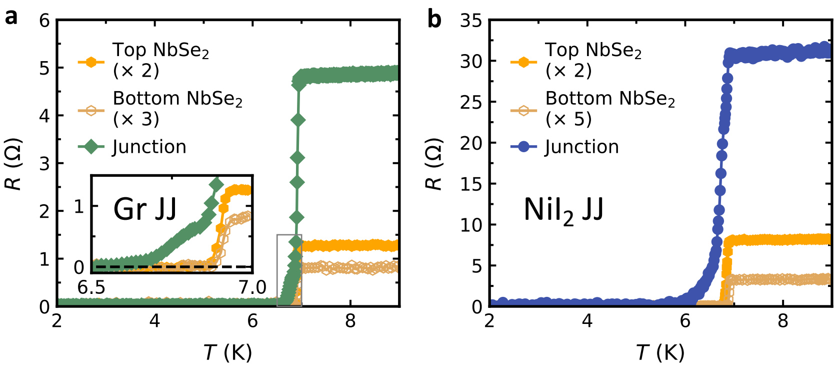

Supplementary Fig. 6 Temperature dependence of the resistance across the junction and flakes. **a**, Four-terminal resistance of the top $NbSe_2$ flake, the bottom $NbSe_2$ flake, and across the junction in the Gr JJ. The inset shows the resistance near the superconducting transition of $NbSe_2$ (marked by the gray square). **b**, Four-terminal resistance of top $NbSe_2$ flake, bottom $NbSe_2$ flake, and across the junction in the NiI_2 JJ. In both JJs, the resistance of both top and bottom $NbSe_2$ flakes sharply drops to zero at the transition temperature ~ 7 K [18], while the junction resistance drops to zero at a lower temperature, consistent with the typical behavior of JJs [19, 20]. It is noted that the resistance of both JJs decreases monotonically as the temperature approaches the transition temperature, suggesting the high quality of our device compared to a non-homogeneous superconductor which would show an anomalous peak above the transition temperature [21].

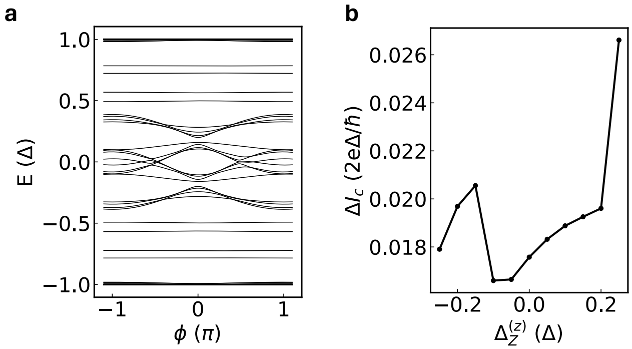

Supplementary Fig. 7 Additional simulations of a helimagnet JJ. **a**, Andreev bound state spectrum of the helimagnet JJ with CPR corresponding to Fig. 4c of the main text with RSOC for $\mathbf{q} \parallel \mathbf{x}$. **b**, ΔI_c as a function of $\Delta_Z^{(z)}$ (external Zeeman spin-splitting along $\mathbf{z}$ in Fig. 4a of the main text) with a mixed even-odd functional dependence. Parameters used in simulations are: $\Delta = 0.4t$, $\mu = 1.57t$, $\alpha_R = 0.004ta$, $J_{exc} = 0.3t$, $U_{barrier} = 4t$, $|\mathbf{q}| = 0.01\frac{\pi}{a}$, $L_{x,s} = 300a$, $L_{x,n} = 3a$, $L_y = 10a$, and $\xi_{exc} = 5a$ where $t = \frac{\hbar^2}{2m^*a^2}$ and a is the tight binding lattice constant.

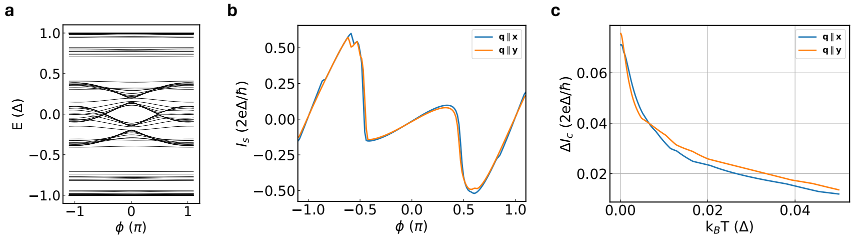

Supplementary Fig. 8 Simulation of a wide helimagnet JJ. Simulation of a wide helimagnet JJ with $L_y = 20a$. **a**, Andreev bound state spectrum for $\mathbf{q} \parallel \mathbf{x}$ with RSOC. **b**, Current-phase relationships. and **c**, Temperature dependence of ΔI_c.

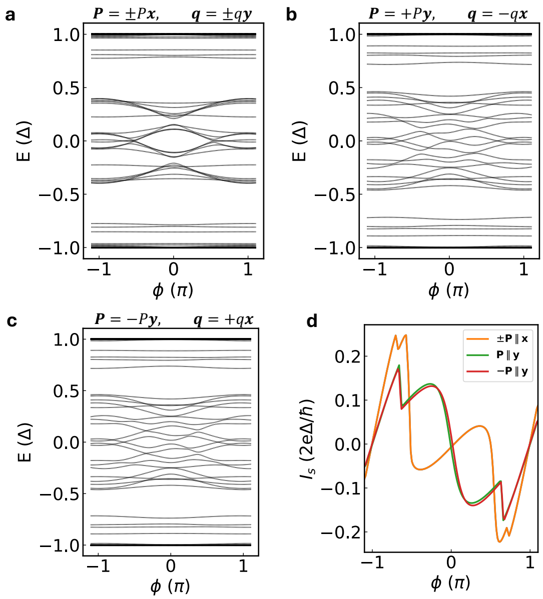

Supplementary Fig. 9 Simulations of multiferroic JJs. a, Andreev bound state spectra of multiferroic JJs for $\mathbf{P} = \pm P\mathbf{x}$ and **b-c** $\mathbf{P} = \pm P\mathbf{y}$ with $\mathbf{P} \times \mathbf{q} \parallel +\mathbf{z}$ (see Fig. 4a of the main text). **d**, CPRs of multiferroic JJs calculated from their corresponding spectra shown in panel **a-c**.

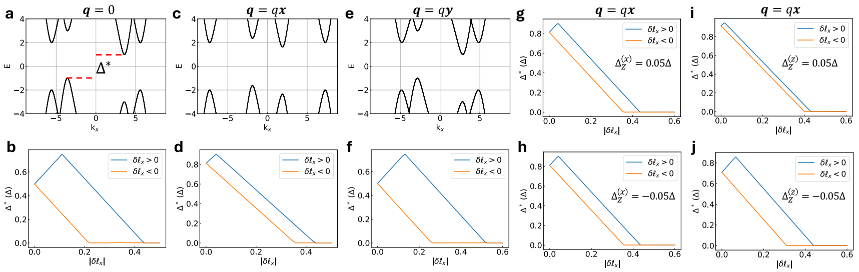

Supplementary Fig. 10 Single-ℓ helical superconductivity with a helimagnet. Dispersion with $\ell_x = \ell_{x,0}$ and gap dependence on depairing momentum $\delta\ell_x = \ell_{x,0} - \ell_x$, where $\ell_{x,0}$ is the equilibrium momentum, for a **a-b**, helical Rashba superconductor, **c-f**, helimagnet superconductor with Rashba spin-orbit coupling. **g-h**, Spectral gap suppression with depairing momentum for positive (top) and negative (bottom) Zeeman splitting along the x-direction. **i-j**, Spectral gap suppression with depairing momentum for positive (top) and negative (bottom) Zeeman splitting along the z-direction.

References

[1] Klein, D. R., MacNeill, D., Lado, J. L., Soriano, D., Navarro-Moratalla, E., Watanabe, K., Taniguchi, T., Manni, S., Canfield, P., Fernández-Rossier, J. & Jarillo-Herrero, P. Probing magnetism in 2D van der Waals crystalline insulators via electron tunneling. *Science* **360**, 1218-1222 (2018).

[2] Song, Q., Occhialini, C. A., Ergeçen, E., Ilyas, B., Amoroso, D., Barone, P., Kapeghian, J., Watanabe, K., Taniguchi, T., Botana, A. S., Picozzi, S., Gedik, N. & Comin, R. Evidence for a single-layer van der Waals multiferroic. *Nature* **602**, 601-605 (2022).

[3] Kim, J. H., Jung, T. S., Lee, Y., Kim, C., Park, J.-G. & Kim, J. H. Terahertz evidence of electromagnon excitations in the multiferroic van der waals insulator NiI_2. *Phys. Rev. B* **108**, 064414 (2023).

[4] Kurumaji, T., Seki, S., Ishiwata, S., Murakawa, H., Kaneko, Y. & Tokura, Y. Magnetoelectric responses induced by domain rearrangement and spin structural change in triangular-lattice helimagnets NiI_2 and CoI_2. *Phys. Rev. B* **87**, 014429 (2013).

[5] Ferrell, R. A. & Prange, R. E. Self-field limiting of Josephson tunneling of superconducting electron pairs. *Phys. Rev. Lett.* **10**, 479 (1963).

[6] Yamashita, T. & Onodera, Y. Magnetic-field dependence of Josephson current influenced by self-field. *J. Appl. Phys.* **38**, 3523-3525 (1967).

[7] Stuehm, D. L. & Wilmsen, C. W. Diffraction patterns and vortex structure of asymmetrical and cross Josephson junctions. *J. Appl. Phys.* **45**, 429-433 (1974).

[8] Tinkham, M. *Introduction to Superconductivity* (Dover Publications, Mineola, 2004).

[9] Yabuki, N., Moriya, R., Arai, M., Sata, Y., Morikawa, S., Masubuchi, S. & Machida, T. Supercurrent in van der Waals Josephson junction. *Nat. Commun.* **7**, 10616 (2016).

[10] Idzuchi, H., Pientka, F., Huang, K.-F., Harada, K., Gul, O., Shin, Y. J., Nguyen, L. T., Jo, N. H., Shindo, D., Cava, R. J., Canfield, P. C. & Kim, P. Unconventional supercurrent phase in Ising superconductor Josephson junction with atomically thin magnetic insulator. *Nat. Commun.* **12**, 5332 (2021).

[11] Kang, K., Berger, H., Watanabe, K., Taniguchi, T., Forró, L., Shan, J. & Mak, K. F. Van der Waals π Josephson junctions. *Nano Lett.* **22**, 5510-5515 (2022).

[12] Suominen, H. J., Danon, J., Kjaergaard, M., Flensberg, K., Shabani, J., Palmstrøm, C. J., Nichele, F. & Marcus, C. M. Anomalous Fraunhofer interference in epitaxial superconductor-semiconductor Josephson junctions. *Phys. Rev. B* **95**, 035307 (2017).

[13] Nevirkovets, I. P., Evetts, J. E. & Blamire, M. G. Transition from single junction to double junction behaviour in SISIS-type Nb-based devices. *Phys. Lett. A* **187**, 119-126 (1994).

[14] Zhao, S. Y. F., Cui, X., Volkov, P. A., Yoo, H., Lee, S., Gardener, J. A., Akey, A. J., Engelke, R., Ronen, Y., Zhong, R., Gu, G., Plugge, S., Tummuru, T., Kim, M., Franz, M., Pixley, J. H., Poccia, N. & Kim, P. Time-reversal symmetry breaking superconductivity between twisted cuprate superconductors. *Science* **382**, 1422-1427 (2023).

[15] Barone, A., Johnson, W. J. & Vaglio, R. Current flow in large Josephson junctions. *J. Appl. Phys.* **46**, 3628-3632 (1975).

[16] Pippard, A. B. & Shoenberg, D. Trapped flux in superconductors. *Philos. Trans. R. Soc. Lond. A* **248**, 97-129 (1955).

[17] Hou, Y., Nichele, F., Chi, H., Lodesani, A., Wu, Y., Ritter, M. F., Haxell, D. Z., Davydova, M., Ilić, S., Glezakou-Elbert, O., Varambally, A., Bergeret, F. S., Kamra, A., Fu, L., Lee, P. A. & Moodera, J. S. Ubiquitous superconducting diode effect in superconductor thin films. *Phys. Rev. Lett.* **131**, 027001 (2023).

[18] Kershaw, R., Vlasse, M. & Wold, A. The preparation of and electrical properties of niobium selenide and tungsten selenide. *Inorg. Chem.* **6**, 1599-1602 (1967).

[19] Pal, B., Chakraborty, A., Sivakumar, P. K., Davydova, M., Gopi, A. K., Pandeya, A. K., Krieger, J. A., Zhang, Y., Date, M., Ju, S., Yuan, N., Schröter, N. B. M., Fu, L. & Parkin, S. S. P. Josephson diode effect from Cooper pair momentum in a topological semimetal. *Nat. Phys.* **18**, 1228-1233 (2022).

[20] Qiu, G., Yang, H.-Y., Hu, L., Zhang, H., Chen, C.-Y., Lyu, Y., Eckberg, C., Deng, P., Krylyuk, S., Davydov, A. V., Zhang, R. & Wang, K. L. Emergent ferromagnetism with superconductivity in Fe(Te,Se) van der Waals Josephson junctions. *Nat. Commun.* **14**, 6691 (2023).

[21] Vaglio, R., Attanasio, C., Maritato, L. & Ruosi, A. Explanation of the resistance-peak anomaly in nonhomogeneous superconductors. *Phys. Rev. B* **47**, 15302-15303 (1993).
